# Integrated Performance and Capability Analysis of Anticorrosive Cathodic Electrodeposition Coatings: Effect of Polymerization Variables

**DOI:** 10.3390/ma18215051

**Published:** 2025-11-06

**Authors:** Damián Peti, Gabriel Stolárik, Radoslav Vandžura, Miroslav Gombár, Michal Hatala

**Affiliations:** 1 Faculty of Manufacturing Technologies with a Seat in Prešov, Technical University of Kosice, Štúrova st. 31, 080 01 Prešov, Slovakia; radoslav.vandzura@tuke.sk (R.V.); michal.hatala@tuke.sk (M.H.); 2 Faculty of Mechanical Engineering, University of West Bohemia Pilsen, Univerzitní 8, 306 14 Plzeň, Czech Republic; gombar.mirek@gmail.com

**Keywords:** cathodic electrodeposition, coatings, performance indices, ISO grades, EDX analysis, SEM, Design of Experiments, adhesion, impact test

## Abstract

The presented research delivers a comprehensive evaluation of anticorrosive cathodic electrodeposition (CED) coatings through an integrated performance and process capability analysis—an approach that remains extremely limited in the literature, particularly in the context of statistically designed experiments (DoEs) applied to CED systems. This study therefore addresses a notable gap by focusing on the role of polymerization variables in determining coating quality through DoE to quantify the influence on coating thickness uniformity, adhesion integrity and impact resistance, while all other deposition parameters were rigorously controlled. Prior to coating application, all specimens were prepared and conditioned in accordance with ISO 1513:2010. Coating thickness was determined in compliance with ISO 2808:2019, adhesion was characterized by cross-cut methodology according to ISO 2409:2020 and dynamic mechanical resistance was evaluated using a falling-weight apparatus in accordance with ISO 6272-1:2011. The obtained datasets were subjected to statistical capability analysis within the PalstatCAQ environment, providing Cp, Cpk, Pp and Ppk indices in line with ISO 22514-7:2021 and IATF 16949:2016 requirements. Results evidenced non-linear dependencies of thickness formation on curing parameters, with potential capability indices (Cp > 1.8; Pp ≈ 1.4) indicating favorable process dispersion, while performance indices (Cpk < 0.5; Ppk < 0.4) revealed systematic mean shifts and deviations from normality confirmed by Shapiro–Wilk and Anderson–Darling tests. Adhesion testing demonstrated a direct correlation between curing conditions and interfacial bonding, reaching ISO Grade 0 classification. Complementary impact resistance assessments corroborated these findings, showing that insufficient curing induced extensive cracking and delamination. Furthermore, SEM–EDX analysis performed on Sample n.3 of X_2_ variable confirmed the chemical integrity and multilayered structure of the CED coating.

## 1. Introduction

Surface modification has been recognized as a decisive stage in contemporary manufacturing, fundamentally determining the functional properties, durability, and reliability of final components. High precision surface modifications provide protection against mechanical degradation, corrosive environments, and premature failure, while simultaneously enhancing adhesion for subsequent technological operations [1]. Since corrosion of metallic materials continues to impose substantial economic, environmental, and safety-related challenges, research efforts and industrial practice converge on preventive measures [2]. Organic coatings, applied to metallic substrates, have been widely recognized as an effective barrier against electrochemical degradation; however, their protective efficiency is conditioned by continuity of the coating phase, microstructural homogeneity, and sufficient layer thickness [3]. Among coating technologies, cathodic electrodeposition offers distinct advantages over anodic deposition in terms of corrosion protection. In contrast to anodic processes, where water electrolysis at the anode generates hydrogen ions and oxygen, potentially undermining barrier effectiveness, cathodic deposition allows for more uniform and adherent coatings with enhanced protective performance [4]. Furthermore, recent developments have highlighted the incorporation of inorganic nanoparticles as nanofillers in polymeric coatings. The uniform dispersion of these nanoparticles within the polymer matrix is critical, as it improves barrier properties, mitigates microstructural defects, and contributes to the overall durability and corrosion resistance of the coating.

Within this context, cathodic electrodeposition has established itself as one of the most relevant coating technologies, primarily due to its capacity to generate uniform, adherent, and corrosion-resistant films under the action of an applied electric field [5]. The versatility of this process explains its wide adoption in automotive and industrial sectors. Nevertheless, the final coating performance is not dictated solely by material chemistry. A decisive role is played by technological parameters, such as polymerization time, curing temperature, bath composition, deposition voltage, and pH, as well as by subsequent post-treatment steps. An inappropriate adjustment of these conditions has been shown to induce internal stresses, cracks, and insufficient adhesion, thus diminishing the long-term reliability of the protective system [6].

Although numerous investigations have addressed these factors, a prevailing trend has been their evaluation in isolation, often restricted to thickness characterization or corrosion performance. What remains underexplored is the interdependence between process parameters, microstructural evolution, and mechanical durability. This research gap underlines the necessity of an integrated methodological framework that not only employs destructive and non-destructive testing but also quantifies process stability through process capability indices. Statistical descriptors such as Cp, Cpk, Pp, and Ppk have been established in process engineering as reliable indicators of variability and compliance with target specifications by Sahu & Maiti [7], yet their systematic application in the evaluation of CED processes is still limited. Their incorporation enables direct correlation between process stability and functional outcomes such as coating thickness, adhesion, and impact resistance, thereby opening pathways toward predictive optimization.

Previous investigations [8] confirmed the decisive role of polymerization parameters, where extending curing time from 20 to 60 min increased the average crack length from 125 µm to 210 µm at elevated temperatures. This demonstrated a direct link between curing kinetics, residual stress formation, and crack propagation. Further studies on the same substrate system emphasized the importance of pretreatment, where higher degreasing temperatures produced measurable increases in layer thickness [9], while optimized concentrations of degreasing agents enhanced adhesion and deposition uniformity [10]. These findings illustrate the dual influence of processing: polymerization primarily governs stress development and crack initiation, whereas pretreatment defines deposition homogeneity and structural continuity.

Complementary perspectives have been provided by Fejko et al. [11], who combined predictive modeling with layer thickness measurements (ISO 2808:2019) [12] and EDX microanalysis. Their results showed that optimized deposition voltage and prolonged deposition time increased coating thickness into the 18–23 µm range and improved compositional stability, while structural equation modeling accounted for over 70% of thickness variability. Similar attention to interfacial behavior was devoted to adhesion, a critical determinant of long-term coating performance. Fejko et al. [13] also demonstrated that 32 aluminum samples subjected to CED exhibited adhesion classifications predominantly between 0 and 1 according to ISO 2409:2020, indicative of excellent coating–substrate bonding. These findings emphasize the necessity of simultaneously evaluating layer thickness and adhesion to establish a mechanistic understanding of coating reliability. Complementary investigations by Titu et al. [14] on Al7175 substrates revealed that cathodic electrocoating significantly enhanced adhesion compared to thermal spray coatings, with improvements on the order of 20%, highlighting the influence of deposition technology on interfacial strength. Further, Parthasarathy et al. [15] systematically demonstrated that mechanical, chemical, and physical surface modifications—such as plasma treatment and ion implantations substantially improve coating adhesion and durability across diverse substrates.

The decisive influence of pretreatment has also been highlighted in the comparative work of Skotnicki and Jędrzejczyk [16], who showed that chemical cleaning (12% HCl) led to thicker, more uniform coatings (≈ 33 µm, s.d. 1.62 µm) compared to abrasive blasting (≈26 µm, s.d. 6.03 µm). Improved surface smoothness (Ra ≈ 0.97 µm vs. 3.45 µm) and lower defect density were reflected in superior adhesion and resistance to delamination, further reinforcing the argument that process variability in pretreatment propagates directly into coating quality. The study involved cathodic electrodeposition at 230–270 V for 3 min followed by curing at 180 °C, revealing that chemically cleaned substrates consistently achieved more compact structures and lower friction coefficients (≈0.13 vs. 0.35) and enhanced corrosion resistance in salt spray tests compared to treated samples.

From the mechanical standpoint, coating durability requires resistance not only to corrosion but also to sudden mechanical loads. Wang et al. [17] highlighted the role of coating–substrate interactions in fatigue and delamination, emphasizing the need for standardized evaluation. Accordingly, the impact resistance test (ISO 6272-1:2011) provides a reproducible measure of performance under dynamic conditions. While most prior research applied this test to hard coatings, its extension to CED systems introduces a novel dimension, particularly when combined with polymerization variability.

By synthesizing these findings, the present study proposes an integrated methodology where process capability indices (Cp, Cpk, Pp, Ppk) are evaluated alongside adhesion, thickness, and impact resistance. This holistic framework not only validates standardized testing protocols but also expands their interpretative capacity by linking functional performance directly to process stability. In doing so, the study advances predictive understanding of CED and establishes a foundation for optimizing both reliability and industrial applicability of cataphoretic coatings.

While Titu et al. [14] and Parthasarathy et al. [15] established predictive links between deposition parameters and coating thickness, Skotnicki and Jędrzejczyk [16] demonstrated the decisive influence of pretreatment on adhesion and homogeneity, and Wang et al. [17] emphasized the mechanical perspective of coating–substrate interaction, these approaches remain fragmented. None of these studies has simultaneously integrated destructive and non-destructive testing with process capability indices, nor directly quantified the relationship between statistical process stability and functional coating performance. The present research addresses this gap by combining adhesion and impact resistance testing with Cp, Cpk, Pp, and Ppk evaluation, thereby establishing a predictive framework that connects process variability with both mechanical durability and coating reliability. This integrated methodology not only consolidates existing knowledge but also introduces a methodological innovation with direct industrial relevance. It enables early identification of regions prone to defect formation, supports preventive interventions in industrial practice, and allows more precise optimization of polymerization conditions compared to traditional empirical approaches. This contribution represents the core novelty of the present study, offering a structured pathway to link process control with coating reliability in real manufacturing conditions.

## 2. Materials and Methods

The present research focused on the systematic implementation of both non-destructive and destructive testing of samples subjected to a planned experiment within the technological process of CED coating. A Design of Experiments (DoE) approach was adopted to efficiently investigate the effects of multiple process parameters, allowing for the simultaneous assessment of individual and interaction effects on the quality of the deposited coatings [18]. This methodology was chosen due to its ability to provide statistically robust and reproducible results while minimizing the number of required experimental runs, offering a clear advantage over traditional one factor at a time approaches.

The experimental factors selected for the study were (curing) polymerization time and polymerization temperature, as these are known to have a significant influence on coating thickness, mechanical properties and corrosion resistance. The chosen factor levels were based on literature data and preliminary experimental observations mentioned in previous research [8,9,10], covering the full range of industrially relevant conditions within the SEM-EDX analysis to verify and correlate the chemical compositions of coatings [11].

Prior to coating application, the test specimens were prepared and conditioned in accordance with ISO 1513:2010 [19], ensuring uniform surface quality and suitability for reproducible coating deposition. All other technological parameters of the CED process, including degreasing solution concentration, degreasing time, deposition voltage, and rinsing steps, were kept constant to isolate the effects of the investigated factors. The process conditions were set to ensure that the resulting coating thickness was representative of typical industrial conditions.

### 2.1. Materials Selection VDA-239-100 CR4

The experimental material was a low-carbon cold-rolled steel in accordance with the VDA 239-100 standard [20], widely employed in the automotive industry. This steel grade, conforming to the specifications of DIN EN 10130 [21], exhibits excellent deep-drawing capacity, resistance to strain aging, and high surface quality, making it particularly suitable as a substrate for CED coatings, where both adhesion and uniformity of coating thickness are critical performance indicators. The homogeneous surface ensures consistent interaction with the applied coating, enhancing the reliability of subsequent measurements and statistical analyses.

Within the framework of the present DoE approach [22], CR4 steel was selected due to its industrial relevance and stable baseline properties (Table 1), which minimize uncontrolled variability and enable reproducible results. These characteristics are essential when integrating non-destructive and destructive evaluation methods, such as layer thickness measurement—ISO 2808:2019 [12], cross-cut adhesion testing—ISO 2409:2020 [23], and impact resistance testing—ISO 6272-1:2011 [24], alongside process capability assessment using Cp, Cpk, Pp, and Ppk indices. By employing a standardized and high-quality substrate, the study ensures that observed variations in coating performance can be reliably attributed to process parameters, thereby supporting predictive insights into process–property relationships.

The favorable formability of CR4 steel additionally enables the investigation of coating behavior on geometrically complex surfaces (Table 2), further supporting its suitability as an experimental substrate. By employing a material—VDA 239-100 CR4—that reflects industrial practice, the results obtained within the DoE framework can be directly associated with real production conditions, thereby increasing the applicability of the findings.

### 2.2. Experimental Setup

The technological process of CED/KTL coating (Table 3) represents a complex surface finishing method in which an inorganic–organic protective layer is uniformly deposited on the substrate via electrophoretic deposition from an aqueous electrolyte. This coating technology is extensively employed in the automotive and mechanical engineering industries, providing high-quality corrosion protection.

Within the experimental framework, the specimens underwent a standardized technological sequence comprising:

- Surface pretreatment, including degreasing and phosphating,
- Subsequent thermal polymerization of the deposited layer.

All phases of the process were conducted under constant conditions, while the key process parameters—polymerization (curing) time and polymerization (curing) temperature—were systematically varied according to the DoE plan. The adopted technological procedure was derived from actual industrial practice, ensuring the relevance and applicability of the experimental results. The primary objective of the experimental setup was to evaluate the influence of the controlled process variables on the final properties of the coating, with particular emphasis on film thickness and mechanical performance.

### 2.3. Design of Experiment

The experimental setup of this study was focused on the systematic investigation of the influence of selected technological parameters of the CED process on the resultant properties of the deposited coating. To ensure objective and reproducible results, DoE methodology was adopted. This approach enables simultaneous analysis of multiple factors and the evaluation of both their individual and interaction effects on the measured responses, while minimizing the total number of experimental runs required to achieve statistically significant conclusions.

This study represents one part of a broader DoE framework that systematically investigates all critical process stages of the industrial KTL line. Within this larger experimental plan, eight key technological parameters (X_1_–X_8_) were analyzed, including surface pretreatment, phosphating, electrodeposition and polymerization. Polymerization was selected as the focus of this work because it represents the final and most critical stage affecting coating quality, mechanical integrity and corrosion protection based on a critical review of the literature and preliminary experimental observations:

- X_1_—Curing time [min], ranging from 13 to 27 min.
- X_2_—Curing temperature [°C], ranging from 150 to 250 °C.

These factors were investigated at five levels each (Table 4), resulting in a total of ten experimental samples, which were divided into two groups to independently evaluate the influence of curing time [min] and curing temperature [°C]. To isolate their individual effects, the curing temperature was fixed at 200 °C during the curing time experiments, representing the nominal process setting previously optimized in earlier studies [8,9,10]. Conversely, when evaluating the influence of curing temperature, the curing time was held constant at 20 min, based on the results of “Kinetic Analysis of Crack Formation in Cathodic Electrodeposited Coatings: Influence of Polymerization Time and Thermal Conditions” [8], where polymerization conditions were optimized according to measured crack length.

This strategy ensured that variations in the measured coating properties could be attributed exclusively to the parameter under investigation, thereby eliminating confounding effects and ensuring factor isolation. All other technological parameters of the process—such as degreasing solution concentration, chemical degreasing time, phosphating time, CED deposition voltage and others—were strictly controlled throughout the entire experiment to ensure a stable baseline and reproducible boundary conditions (Table 5).

The combination of statistically structured experimentation with strictly controlled boundary conditions provides a robust basis for identifying threshold behavior and optimizing key curing parameters. This structure is essential for determining the direct and isolated effects of curing time and curing temperature on coating thickness, mechanical performance and functional stability of the deposited films.

### 2.4. Measurement Methods

The evaluation of the deposited CED coatings was performed through an integrated methodology that combined both non-destructive and destructive testing techniques. This dual approach enabled a comprehensive assessment of the coatings, not only in terms of their basic thickness but also with respect to adhesion quality and mechanical resistance under simulated operational stresses. Particular attention was paid to ensuring methodological rigor, whereby each test was selected to reflect a specific functional property of the coating relevant to industrial performance requirements.

To ensure accuracy and reproducibility, all measurements were carried out using accredited instruments under controlled laboratory conditions at the Technical University of Košice (temperature 23 ± 2 °C and relative humidity 50 ± 5%), outside the CED production line of KTL, s.r.o., with strict adherence to the relevant technical standards. This ensured stable and reproducible measurement conditions, minimizing environmental influences on the results. Non-destructive measurements provided reliable data on layer thickness and uniformity across the coated surfaces, while destructive methods validated adhesion strength and resistance to localized mechanical loading in accordance with the corresponding technical standards:

- Elcometer 456C—coating thickness measurement according to EN 2808:2019 [12]

**Figure 1:**
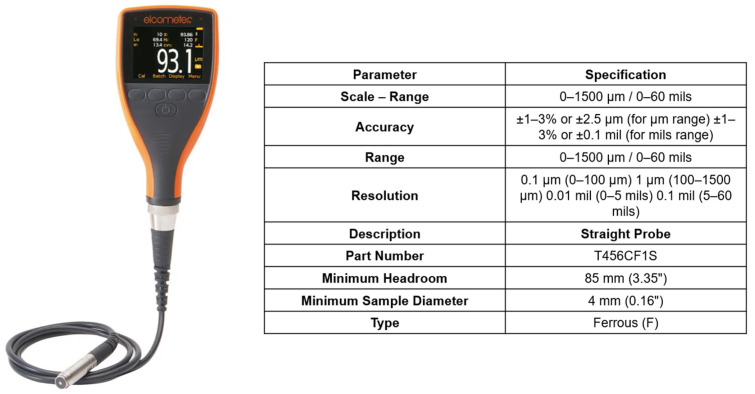
Specification of the Elcometer 456C coating thickness gauge and direct-contact probe type T456CF1S.

The thickness of the applied coating was quantified using a portable Elcometer 456C instrument (Figure 1) by Elcometer Ltd., Manchester, UK, which operates based on a non-destructive measurement principle suitable for evaluating dry films deposited on metallic substrates with different magnetic properties. Accurate measurement outcomes depend on prior instrument calibration, ensuring traceability and correctness of the recorded data. The instrument demonstrates a high degree of metrological accuracy (±1%) and conforms to the requirements specified in international standards for protective coating thickness measurement.

**Figure 2:**
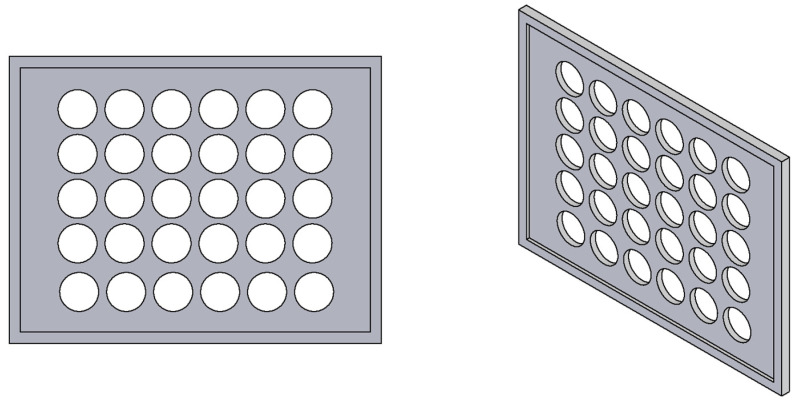
3D-printed fixture for precise and repeatable coating thickness measurement.

For the measurements, a direct-contact probe type T456CF1S (Elcometer Ltd., Manchester, UK) was employed, specifically engineered for non-magnetic coatings on ferromagnetic substrates. The probe features a measurement range of 0–1500 µm and a manufacturer-specified accuracy of ±1–3% or ±2.5 µm, depending on coating thickness and measurement conditions, thus providing reliable and reproducible results across a broad spectrum of industrially relevant layers. To ensure repeatability and uniformity of measurement, a custom-designed auxiliary device was fabricated using 3D printing (Figure 2). This tool enabled precise and consistent positioning of the probe during measurements. For each sample, 30 repeated measurements were performed, providing statistically reliable data and minimizing the influence of random errors.

All measurements were performed in strict accordance with the relevant standard: EN 2808:2019—Paints and varnishes—Determination of film thickness [12]. The standardized methodology ensures that the results are metrologically valid, reproducible, and suitable for subsequent statistical analysis within the DoE framework.

- Elcometer 107–5 SP 3000—evaluation of coating adhesion by cross-cut test in compliance with EN ISO 2409:2020 [23]

**Figure 3:**
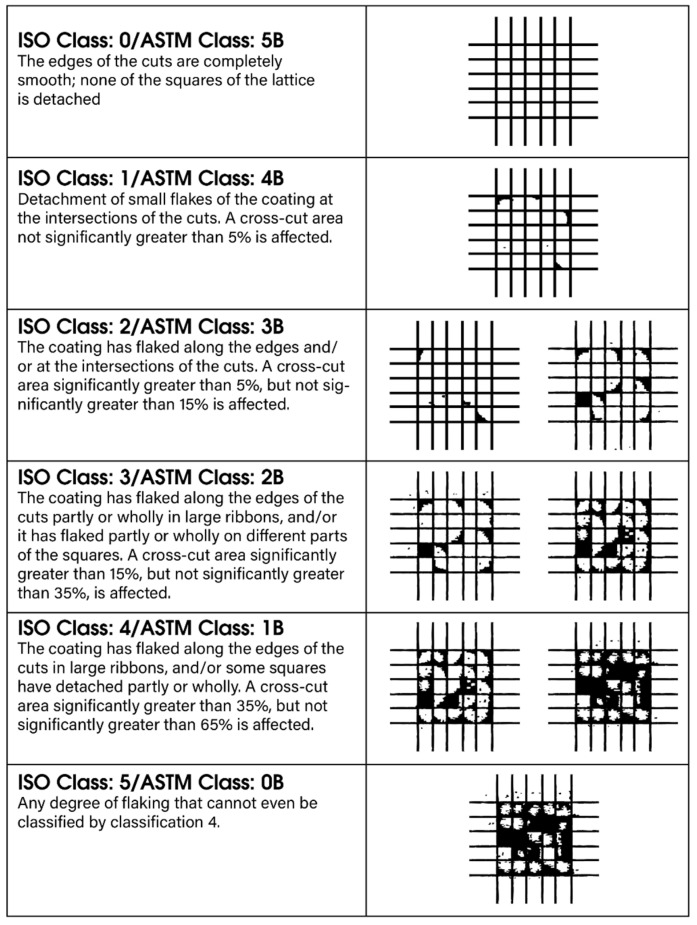
Classification of Cross-Cut Adhesion Test [26].

The adhesion of the coating to the substrate was evaluated using a cross-cut test (Figure 3) in accordance with EN ISO 2409:2020 [23], a standardized procedure for assessing the adhesion of coating systems [25]. The measurements were conducted with an Elcometer 1542 device (Elcometer Ltd., Manchester, UK), recognized for its ease of use and reliable performance across a wide range of surface finishes.

Coating adhesion is strongly influenced by the quality of surface pretreatment, with thorough degreasing of the substrate being particularly critical. The cross-cut test involves creating a series of twelve intersecting cuts—six parallel and six perpendicular forming a uniform grid pattern (Figure 3). Each perpendicular cut extends 1–2 mm beyond the ends of the parallel cuts, ensuring complete intersection of the incisions. The spacing between adjacent cuts is defined by the standard; in this study, a spacing of 1 mm was applied, as prescribed for coatings with a thickness of up to 60 µm.

- TQC SP1880—impact resistance testing according to ISO 6272-1:2011 [24]

**Figure 4:**
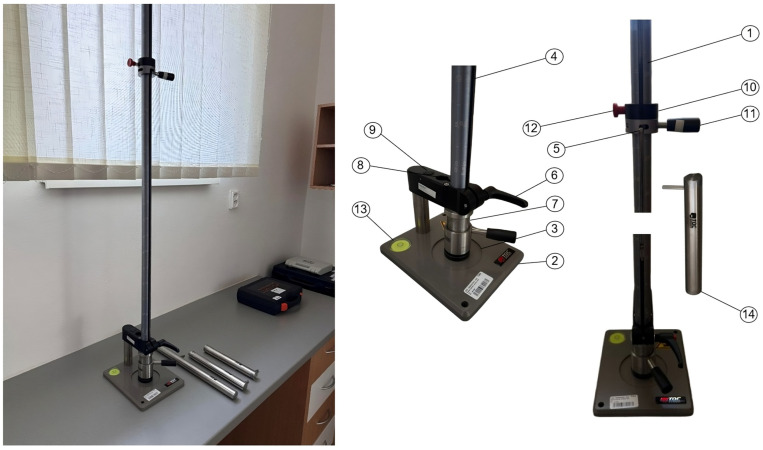
TQC SP1880 device. 1—Guide tube; 2, 6, 13—Base plate assembly with bubble level; 3—Die 16.3 mm, 27 mm; 4—Weight 1 kg; 5—Weight lifting pin; 7—Punch 15.9 mm; 8—Punch 12.7 mm; 9—Lifting pin to release punch; 10, 11, 12—Release collar; 14—Weight witch punch 20 mm [27].

The mechanical resistance of the coating system to dynamic loading was assessed using an impact test (Figure 4), which evaluates both the elastic behavior of the coating and its adhesion to the substrate under sudden deformation. The test was conducted in accordance with ISO 6272-1:2011—Paints and varnishes—Rapid deformation tests (impact resistance)—Part 1: Falling-weight test, large-area indenter [24], employing a TQC SP1880 device (TQC Sheen B.V., Capelle aan den IJssel, The Netherlands) specifically designed to simulate controlled impact loading.

The selection of this testing equipment is justified by the requirements of CED coatings, which serve as a primer layer in automotive and industrial applications and must exhibit sufficient durability against dynamic mechanical stresses. The TQC SP1880 enables reproducible simulation of service-relevant conditions, such as stone chipping, vibration, or accidental impacts, while providing precise control over impact energy. This makes it particularly suitable for correlating variations in polymerization parameters, such as curing temperature and crosslinking density, with the coating’s resistance to cracking, delamination, or loss of adhesion. During the test, a defined impact energy was applied to the specimen using a steel weight with a spherical tip of 20 mm diameter. The weight was released from a precisely specified height and with a defined mass, ensuring reproducible and controlled application of impact force to the coating surface. This methodology provides quantitative information on the coating’s ability to resist short-term mechanical deformation, thereby delivering critical insights into the durability of KTL coatings under service-like dynamic conditions.

## 3. Results

### 3.1. Influence of Curing Parameters on Layer Thickness

To evaluate the influence of curing time (X_1_) on the final thickness of cathodically deposited coatings, a series of measurements was performed on five samples with varying curing durations. For each specimen, 30-point thickness measurements were acquired using the Elcometer 456C.

Table 6 summarizes the descriptive statistics of coating thickness measured at different curing durations. The shortest curing time of 13 min produced the highest mean layer thickness of 18.26 µm, which can be attributed to the presence of residual solvents and incomplete stabilization of the polymer matrix. Conversely, the lowest mean thickness of 14.62 µm was observed at 17 min. Further extension of the curing duration resulted in gradual layer densification and stabilization, reaching a final mean value of 17.47 µm at 27 min. This development indicates a non-linear response of coating thickness to curing time, governed by the competing effects of solvent evaporation, polymer chain mobility, and crosslinking kinetics.

Such behavior reflects the complex interplay between polymerization kinetics and solvent evaporation. Insufficient curing leads to incomplete crosslinking and residual moisture in the film, whereas prolonged curing enhances polymer chain mobility and coalescence, producing thicker but more heterogeneous layers.

**Figure 5:**
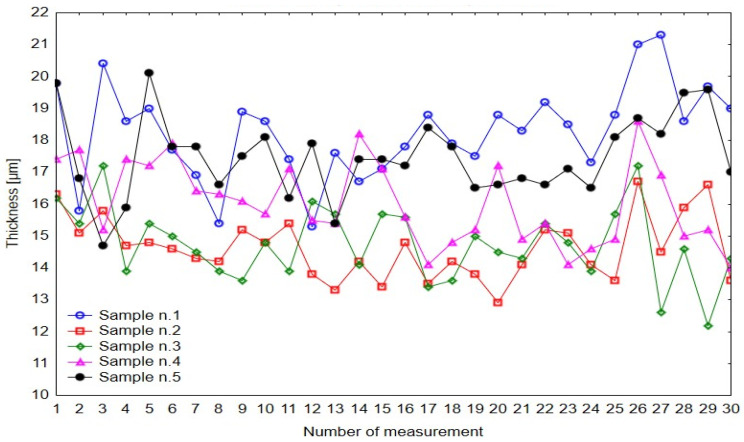
Effect of Curing Time [min] on Dry Coating Thickness [µm].

The curing time interval of 13–27 min (factor levels −2.05464 to +2.05464) was systematically evaluated to characterize its effect on layer growth dynamics (Figure 5). Thickness values ranged from 11 µm to 22 µm, confirming noticeable spatial heterogeneity of the deposited film. Sample 1 exhibited the highest mean thickness but also the largest dispersion (Std. Dev = 1.455 µm), indicating incomplete process stabilization caused by solvent retention and irregular film coalescence. Samples 2 and 3 showed lower mean thickness values (14–15 µm) with narrower standard deviations (0.983 µm and 1.178 µm), reflecting a more stable and uniform deposition regime. Samples 4 and 5 exhibited intermediate mean values and moderate variability, indicating progressive stabilization of the coating structure at extended curing durations.

**Figure 6:**
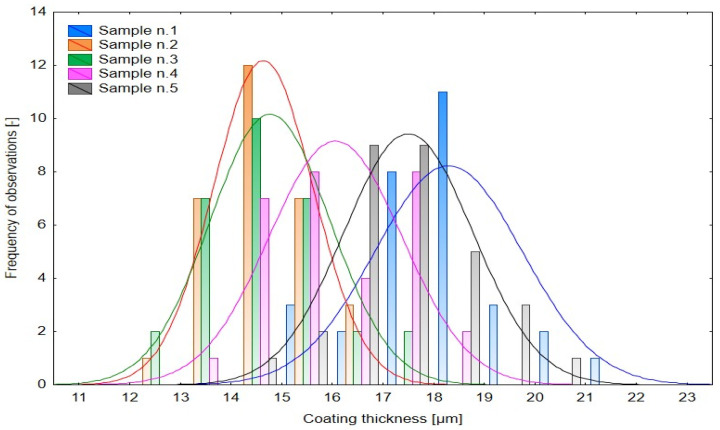
Histogram of multiple variables—Influence of Curing Time on Layer Thickness.

The histogram distributions (Figure 6) confirm a systematic shift in modal values with curing time. Short curing durations yielded thinner and more uniform coatings, whereas extended durations led to thicker layers with broader distributions. This response is typical for cataphoretic coatings, where film growth is driven by the combined kinetics of polymer crosslinking and solvent transport.

**Figure 7:**
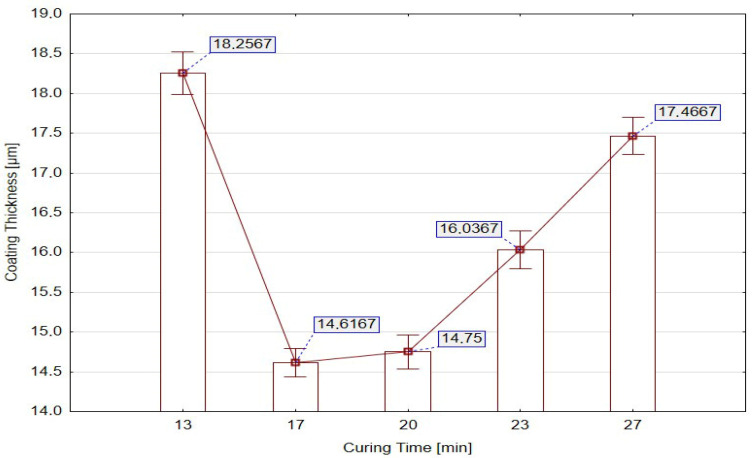
Mean coating thickness with standard error for individual curing durations [min].

Figure 7 provides a complementary statistical visualization of the mean coating thickness with standard error for each curing duration. At 13 min, the highest mean thickness was accompanied by a wide error bar, evidencing unstable film formation. In contrast, at 17 and 20 min, both the mean thickness and the variability decreased significantly, indicating an optimal balance between solvent removal and network consolidation. Beyond 23 min, the increase in both mean thickness and error bar width indicates the onset of heterogeneous film growth, likely caused by secondary coalescence and increased molecular mobility within the polymer structure. The X_1_ factor (curing time) exerts a direct, statistically significant, and technologically decisive influence on coating thickness. Short curing durations (13–17 min; factor levels −2.05464 to −1) consistently produced thinner but more uniform coatings, indicating stable process conditions. Optimized curing durations (20–27 min; factor levels 0 to +2.05464) resulted in thicker coatings, yet at the cost of greater variability and localized non-uniformities. This clearly demonstrates the threshold behavior typical of thermally activated film formation.

The evaluation of the collected experimental data was carried out using the PalstatCAQ software environment (version 2025.02.002), which provides advanced tools for statistical process control and quality assessment in accordance with the requirements of IATF 16949:2016 standard [28]. Beyond the direct evaluation of coating thickness, the process was further characterized using statistical capability indices Cp, Cpk, Pp, and Ppk. These indices are widely applied in manufacturing quality control, as they provide a standardized measure of both process variability and centering with respect to specification limits. While Cp and Pp describe the potential capability of the process in terms of spread, the indices Cpk and Ppk additionally reflect the actual positioning of the process mean within the tolerance range from 15 µm to 30 µm.

The process capability analysis revealed a clear discrepancy between potential and actual performance. The potential capability indices reached Cp = 2.29 (2.00–2.57) and Pp = 1.44 (1.26–1.61), indicating that the coating process is, in principle, able to achieve the required tolerance range. However, the performance indices were substantially lower (Cpk = 0.34, Ppk = 0.22), suggesting a significant shift in the process mean towards one of the specification limits. This imbalance results in a high probability of producing non-conforming thickness values despite the sufficient process spread. Such findings emphasize the necessity for process centering and optimization of curing conditions to fully exploit the inherent capability of the coating system.

The comparative evaluation of capability indices according to 22514-7:2021 [29] across the four measurement levels (L1–L4) confirmed the earlier observation of a pronounced discrepancy between potential and actual performance (Table 7). The Cp values (≈2.03–2.04) indicate a process with a theoretically high capability, whereas the corresponding Cpk values (≈0.33–0.36) remain well below unity, confirming a persistent shift in the mean relative to the specification limits. Similarly, the Pp indices (≈1.43–1.44) show a moderate long-term performance, while the Ppk indices (≈0.28–0.36) highlight an insufficient centering of the process. In practical terms, these results imply that although the process variability is adequately controlled (low σ, ranging between 0.199–0.216), the alignment of the process mean with the target value is poor, leading to a higher probability of producing out-of-specification coating thicknesses. The ratios Ppk/Cpk (≈0.63–0.87) and Pp/Cp (≈0.70–0.72) further support this interpretation, as they underline that the real, long-term process performance is significantly lower than its potential capability.

**Figure 8:**
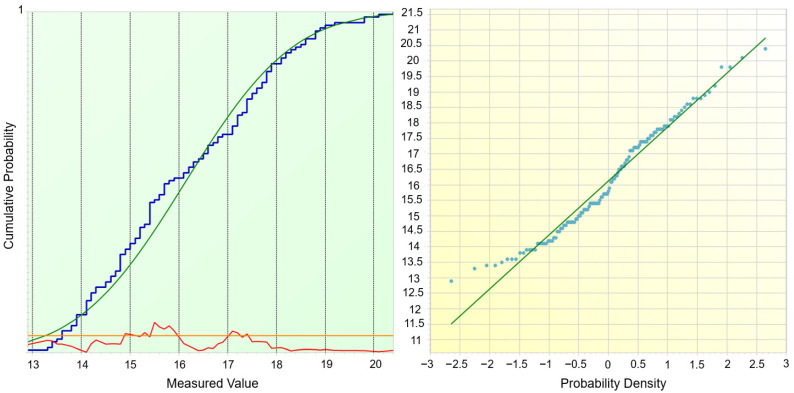
Normality Test—Cumulative Distribution and Q–Q Plot—Curing Time.

The distribution analysis (Figure 8) provides further evidence supporting the observed mismatch between potential and real process capability. The cumulative probability plot (left) shows that the empirical distribution (blue curve) deviates slightly from the fitted normal distribution (green curve), particularly in the lower and upper tails. This deviation is reflected in the residuals (red curve), which remain within acceptable limits but indicate a moderate asymmetry. The probability plot (right) confirms this behavior: although most data points align with the theoretical straight line, systematic deviations can be observed at both extremes, suggesting that the dataset is not perfectly normally distributed. Such departures from normality directly affect the interpretation of capability indices. While Cp and Pp values suggest a process with sufficient spread relative to specification limits, the lower Cpk and Ppk indices reveal the impact of distribution skewness and mean shift. In practical terms, this means that the process generates coating thicknesses with relatively low variability, yet the centering around the nominal target is insufficient, resulting in a significant share of values being closer to or outside the tolerance boundaries.

The statistical evaluation of normality confirmed the graphical observations. The X^2^ test (*p* = 0.5078) and Kolmogorov–Smirnov test (*p* = 0.1361) do not reject the null hypothesis of normality at the 5% significance level, suggesting that the data distribution can be approximated by a Gaussian model. However, more sensitive tests, namely the Shapiro–Wilk (*p* = 0.007276) and Anderson–Darling (*p* = 0.004174), indicated statistically significant deviations from normality. This dual outcome highlights an important aspect: although the distribution may appear sufficiently normal for practical engineering evaluation, subtle deviations (particularly in the tails, as seen in the probability plot) influence the accuracy of capability indices. Specifically, the lower values of Cpk (0.34) and Ppk (0.22) compared with Cp (2.29) and Pp (1.44) reflect this departure, as they capture not only the process spread but also the skewness and mean shift of the data.

**Figure 9:**
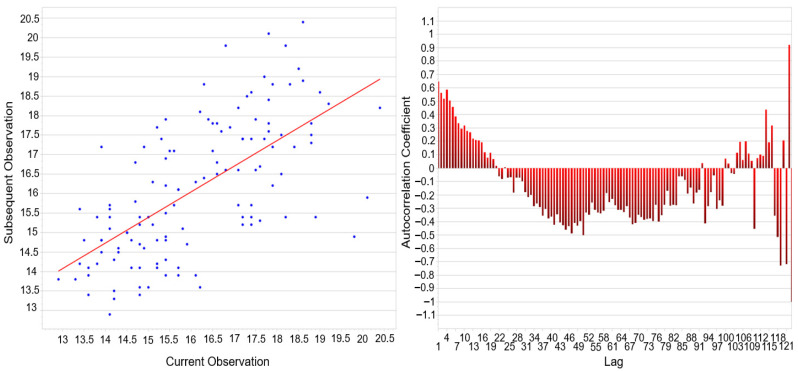
Autocorrelation Analysis—Scatter Plot of Consecutive Observations and Correlogram–Curing Time.

The autocorrelation analysis (Figure 9) confirmed that curing time plays a decisive role in the temporal stability of coating thickness formation. The scatter plot revealed a clear linear dependence between consecutive measurements, indicating that the process retains a “memory” of previous states. This effect was further supported by the correlogram, where short lags exhibited strong positive coefficients, demonstrating a pronounced internal dependence at shorter curing times. With increasing lag, coefficients shifted into negative values, suggesting cyclic or oscillatory behavior likely associated with heat transfer dynamics and the chemical reaction response during curing. At longer lags, correlations re-emerged periodically, indicating a long-term structural component of process variability. These findings provide an explanation for why normality tests (Shapiro–Wilk, Anderson–Darling) rejected the hypothesis of Gaussian distribution and why the capability indices Cpk and Ppk remained low despite favorable Cp and Pp values. The persistence of autocorrelated structures confirms that the curing process is governed by deterministic mechanisms rather than purely random fluctuations, which must be accounted for in process design and control. The process thus exhibits sufficient potential capability, but its real performance is limited by time-dependent dependencies induced by curing time. Consequently, optimization of this parameter is crucial not only for achieving uniform coating thickness but also for eliminating systematic deviations and enhancing real process capability.

To evaluate the influence of curing temperature (X_2_) on the final thickness of cathodically deposited coatings, five samples were prepared with curing temperatures ranging from 150 °C to 250 °C (Table 8).

The measured coating thickness exhibited a distinct non-linear relationship with curing temperature. The mean values ranged from 15.71 µm at 150 °C to 18.52 µm at 200 °C, where the maximum thickness was achieved. This peak corresponds to an optimum thermal activation window, in which the polymer network undergoes intense crosslinking and coalescence, accompanied by efficient solvent evaporation. At low temperatures (150–176 °C), the reduced molecular mobility, slower reaction kinetics, and incomplete solvent removal limit layer growth and lead to thinner, relatively homogeneous coatings. The polymer chains remain in a partially mobile state, and residual moisture and solvent content prevent complete film densification. At the optimal curing temperature of 200 °C, accelerated polymerization reactions result in an increased degree of crosslinking, forming a dense and cohesive structure. In this regime, the polymer viscosity reaches a critical level at which surface leveling and film consolidation occur almost simultaneously. The mobility of the polymer chains allows redistribution and compaction of the coating, leading to a maximum dry film thickness. This is also accompanied by the largest observed variability (Std. Dev = 1.850 µm), indicating localized fluctuations within the layer due to rapid reaction kinetics and spatially non-uniform heat transfer. When curing temperature exceeds the optimal range (224–250 °C), the mean thickness decreases, despite a high level of polymerization. This behavior is characteristic of over-curing phenomena, where excessive thermal energy accelerates solvent flash-off and induces internal stresses caused by differential shrinkage.

**Figure 10:**
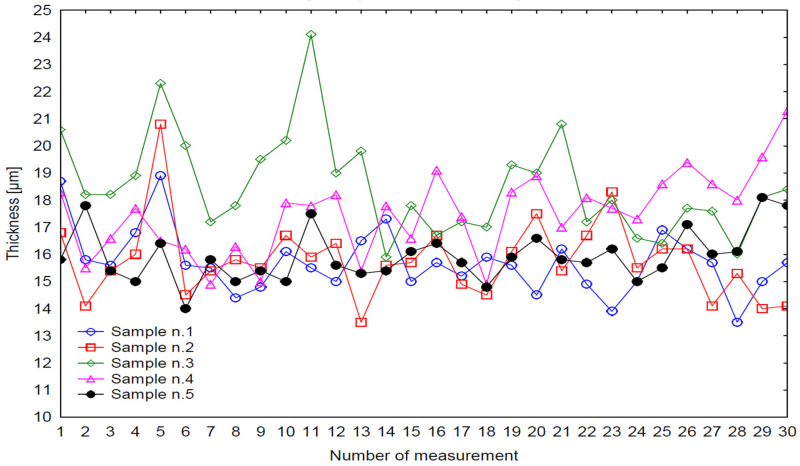
Effect of Curing Temperature [°C] on Dry Coating Thickness [µm].

The distribution of thickness values further substantiates these trends (Figure 10). Across all specimens, values ranged between approximately 11 and 25 µm, confirming the high sensitivity of the cataphoretic process to thermal conditions. Sample 1 displayed a narrow distribution (Std. Dev = 1.186 µm), corresponding to stable but kinetically limited curing. Sample 3 exhibited a broad distribution and increased heterogeneity, while Sample 5 showed the narrowest variability (Std. Dev = 0.960 µm), indicative of an over-cured and stabilized structure with reduced overall thickness.

**Figure 11:**
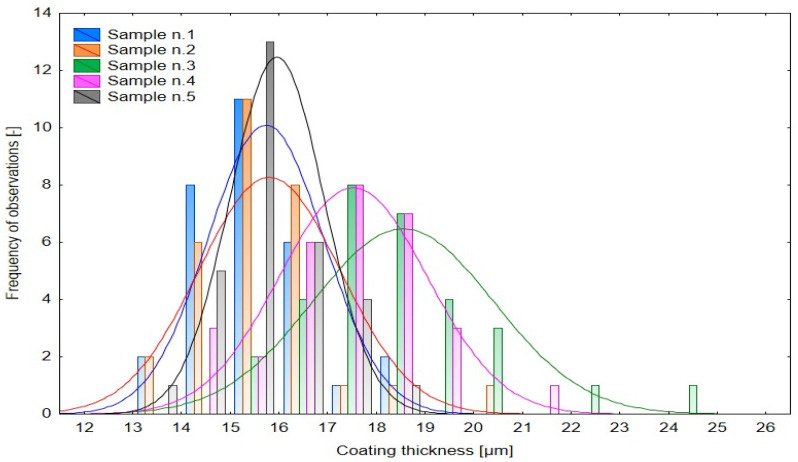
Histogram of multiple variables—Influence of Curing Temperature on Layer Thickness.

The histogram distributions (Figure 11) reveal systematic shifts of the modal peak as curing temperature changes. At lower temperatures, the peak is positioned around 15–16 µm, reflecting uniform but thin layers. At 200 °C, the peak shifts toward 18–19 µm, marking the optimum film-building conditions. Above this threshold, modal values decline again, signaling the onset of thermal degradation processes and diminished deposition efficiency.

**Figure 12:**
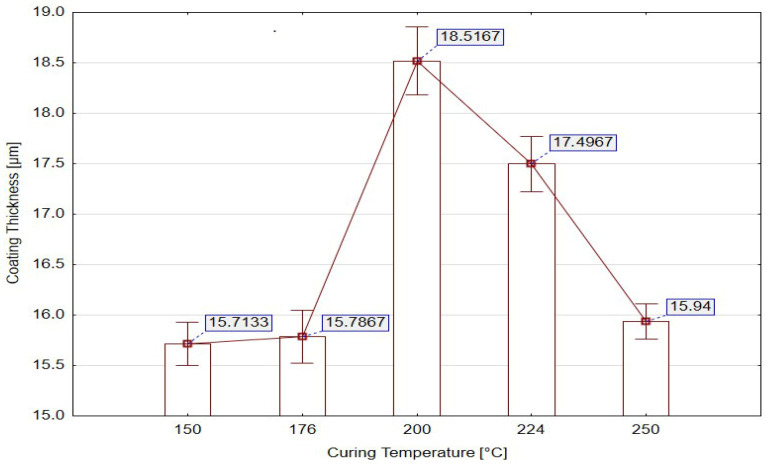
Mean coating thickness with standard error for individual curing temperature [°C].

The mean thickness plot with standard error (Figure 12) provides a clear statistical synthesis of these phenomena. The highest mean thickness and error were recorded at 200 °C, reflecting intensified film growth accompanied by local fluctuations. Below this temperature, the coatings remained thinner with lower variability, indicating uniform deposition but insufficient crosslinking. Above 224 °C, film thickness decreased while variability partially persisted, confirming the unstable character of the over-cured structure. These findings confirm that curing temperature acts as a threshold parameter that directly governs the interplay between film growth, solvent evaporation, polymer chain mobility, and crosslinking kinetics. Below the optimal temperature, the system remains under-cured, with insufficient chain mobility and solvent retention. Within the optimal window (around 200 °C), the reaction kinetics and transport processes are balanced, enabling maximum film build-up and good structural integrity. Exceeding this window results in rapid solvent flash-off, stress development and partial degradation of the layer.

The process capability analysis yielded Cp values in the range of 1.62–2.08, indicating that the inherent variability of the process is sufficiently narrow with respect to the specification limits. However, the corresponding Cpk values (0.36–0.46) were significantly below the acceptable threshold of 1.0, clearly evidencing that the process is not properly centered. A similar trend was observed for the short-term indices, where Pp reached 1.23–1.58, while Ppk remained as low as 0.27–0.35. These results confirm that although the process exhibits a favorable potential capability, its actual performance is strongly limited by systematic shifts away from the nominal value. The discrepancy between Cp and Cpk (as well as between Pp and Ppk) emphasizes that the dominant source of non-conformity is not excessive dispersion, but a lack of centering and process stability. Therefore, corrective actions should focus on re-centering the process and ensuring uniform conditions across production batches rather than solely reducing variability.

The comparative analysis of capability indices (Table 9) across the four measurement levels (L1–L4) according to 22514-7:2021 [29] under varying curing temperatures revealed a similar divergence between potential and actual performance. The Cp values (≈1.80–1.85) consistently exceeded the threshold of 1.33, confirming that the process has a strong potential to meet specification requirements if properly centered. However, the corresponding Cpk values (≈0.30–0.32) remained far below unity, indicating a significant misalignment of the process mean relative to the tolerance window. In parallel, the Pp indices (≈1.40–1.41) demonstrated only moderate long-term capability, while the Ppk indices (≈0.30–0.32) further emphasized the lack of centering and the presence of systematic variation. These results clearly suggest that, although curing temperature provides adequate process spread, the inability to stabilize and align the mean coating thickness with specification limits undermines actual performance. Consequently, precise optimization of curing temperature is essential to minimize mean shifts and fully exploit the inherent capability of the electrodeposition process.

**Figure 13:**
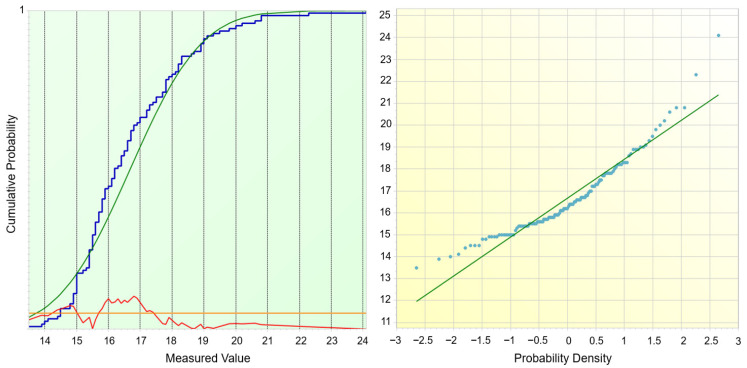
Normality Test—Cumulative Distribution and Q–Q Plot—Curing Temperature.

Figure 13 shows the results of the goodness-of-fit analysis for the measured data. The left panel illustrates the empirical cumulative distribution function (blue line) compared with the theoretical cumulative distribution of the normal distribution (green line). The deviation curve (red line) emphasizes systematic differences between the empirical and theoretical distributions. The right panel presents the Q–Q plot, where the experimental quantiles are plotted against the theoretical normal quantiles. Although values in the central region approximately follow the reference line, significant deviations are observed at both distribution tails, confirming non-normality.

The applied statistical tests further support this finding: the X^2^ test (*p* = 0.0002227), the Kolmogorov–Smirnov test (*p* = 0.00002796), the Shapiro–Wilk test (*p* = 0.000007796), and the Anderson–Darling test (*p* = 0.00002666) all yielded *p*-values far below the significance threshold of 0.05. Therefore, the null hypothesis of normality was rejected with high statistical confidence, indicating that the dataset significantly departs from a Gaussian distribution. This outcome must be accounted for in subsequent processes, capability evaluations and statistical analyses.

**Figure 14:**
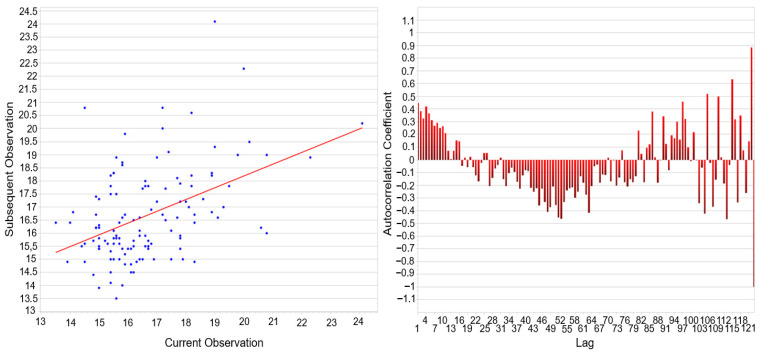
Autocorrelation Analysis—Scatter Plot of Consecutive Observations and Correlogram–Curing Temperature.

Figure 14 illustrates the results of the autocorrelation analysis performed on the same dataset previously subjected to the normality tests (Figure 13). The scatter plot of consecutive observations (x_t_ vs. x_t+1_) suggests a weak positive linear dependency, as indicated by the orientation of the regression line. Ideally, in the case of fully independent and normally distributed data, the scatter should be randomly dispersed without a detectable trend.

However, the observed structure implies that successive values are not entirely independent, which corroborates the findings of the goodness of analysis. The correlogram further confirms this tendency: although most autocorrelation coefficients oscillate around zero, systematic deviations are present, particularly negative correlations in the mid-range and pronounced fluctuations at higher lags. Such patterns indicate that the measurement sequence may be affected by latent process dynamics or uncontrolled disturbances, preventing the dataset from achieving the characteristics of white noise. In combination with the results of the Kolmogorov–Smirnov, Shapiro–Wilk, Anderson–Darling, and X^2^ tests (all yielding *p* < 0.001), these findings collectively demonstrate that the measured variable exhibits both non-normality and serial dependence.

### 3.2. Assessment of Coating Adhesion

The adhesion of the cathodic electrodeposited coatings to the metallic substrate was investigated using the cross-cut method in accordance with ISO 2409:2020 [23]. A standardized multi-blade cutting tool with a spacing of 1 mm was employed to create a lattice pattern consisting of 25 squares in the test area.

The cuts were performed through the coating layer down to the substrate according to the coating thickness and hardness as follows:

- 0–60 μm: 1 mm spacing for hard coatings.
- 0–60 μm: 2 mm spacing for soft coatings.
- 61–120 μm: 2 mm spacing for both hard and soft coatings.
- 121–250 μm: 3 mm spacing for both hard and soft coatings.

**Figure 15:**
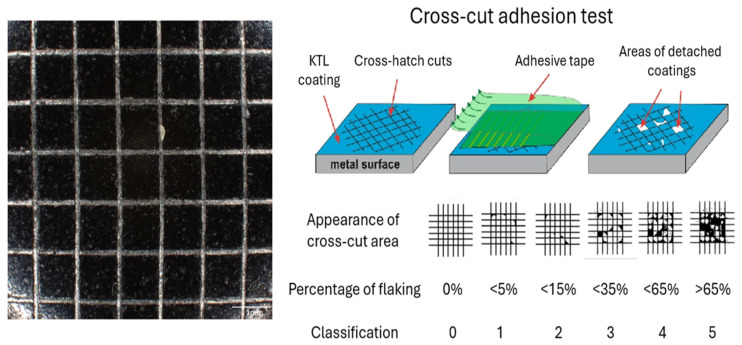
Cross-cut adhesion test of cathodic electrodeposited coating (ISO 2409:2020).

A pressure-sensitive adhesive tape was subsequently applied over the incision area, pressed firmly to ensure proper adhesion, and then removed at a controlled angle and speed, as prescribed by the standard (Figure 15).

The degree of coating detachment was evaluated using the ISO classification scale ranging from Grade 0 to Grade 5. Grade 0 represents intact adhesion with no visible detachment, whereas Grade 5 corresponds to extensive delamination and coating loss within the grid area. Intermediate grades (1–5) reflect progressive levels of detachment along the cut edges or intersections. The classification system employed in this study is summarized in Figure 3 and Figure 15 in accordance with ISO 2409:2020.

Microscopic inspection of the cross-cut areas was performed using the INSPECTIS digital optical system (Pro v3.5—Inspectis AB, Stockholm, Sweden), which enabled high-resolution documentation of the coating response at the incisions. For each specimen, four independent grids (A, B, C and D) were prepared to enhance reproducibility and minimize local variability in adhesion assessment (Table 10 and Table 11). Consequently, every specimen contained four replicated cross-cut tests. For the factor X_1_—influence of curing time on cross-cut test results—this corresponded to a total of 20 evaluations, while for the factor X_2_—influence of curing temperature on cross-cut test results—another 20 evaluations were performed under identical conditions.

The obtained results revealed a clear dependence of adhesion on curing conditions. Specifically, coatings subjected to shorter curing times exhibited higher classification grades (≥3), indicating poor adhesion and partial delamination at the cut intersections. In contrast, prolonged curing times led to significant improvement, with samples achieving Grade 0 or 1, demonstrating strong interfacial bonding and negligible coating detachment. A similar trend was observed with respect to curing temperature. Lower curing temperatures resulted in weaker adhesion, as evidenced by higher grades and pronounced coating removal along the cross-hatched area. Optimized thermal curing, however, yielded predominantly Grade 0 outcomes, reflecting excellent adhesion stability.

Overall, these findings confirm that both curing time and temperature are decisive parameters controlling the adhesion strength of cathodic electrodeposited coatings. The cross-cut test provided a sensitive means of distinguishing between insufficiently cured and optimally cured systems. Nevertheless, since this method primarily assesses static adhesion at localized sites, it was complemented by the impact resistance test, which captures the coating’s performance under dynamic loading. The combination of both approaches therefore delivers a more comprehensive characterization of the coating integrity and durability.

### 3.3. Impact Resistance Test

The resistance of the deposited coatings to dynamic mechanical loading was investigated by means of an instrumented impact test—TQC SP1880. The experiment was conducted in accordance with the general principles of ISO 6272-1:2011 and ASTM D2794 [30], employing a falling-weight apparatus designed to generate controlled impact energy. A hardened steel indenter with a spherical tip of 20 mm in diameter and a total mass of 4.0 kg was released from a height of 1000 mm, providing reproducible short-term mechanical deformation of the coated surface.

The impact energy E imparted to the system is determined as the product of the mass m, the gravitational acceleration g, and the drop height h (1):

(1) E=m×g×h

Substituting the experimental parameters yields (2):

(2) $$E=4.0\;kg\times 9.81\;m\cdot s^{-2}\times 1.0\;m\;\approx 39.2\;J$$

The velocity of the indenter at the moment of contact is expressed as (3):

(3) $$v\;=\;\sqrt{2gh}=\;\sqrt{2\times 9.81\times 1.0}\;\approx \;4.43\;m\times s^{-1}$$

Consequently, the momentum transmitted to the coating–substrate system is (4):

(4) $$p=m\times v=4.0\times 4.43\approx 17.7\;kg\times s^{-1}$$

**Figure 16:**
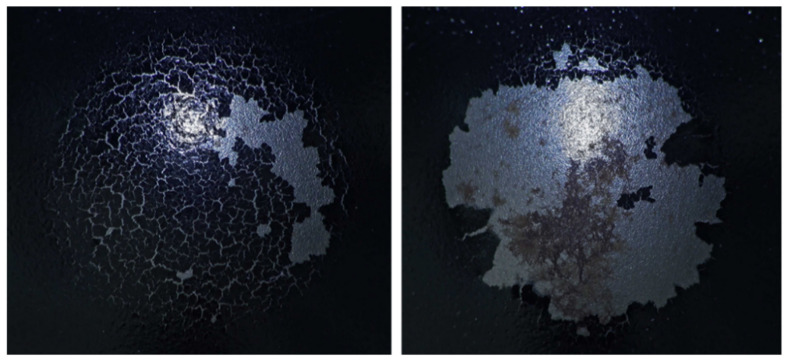
Heavily Damaged Sample (Factor −1).

**Figure 17:**
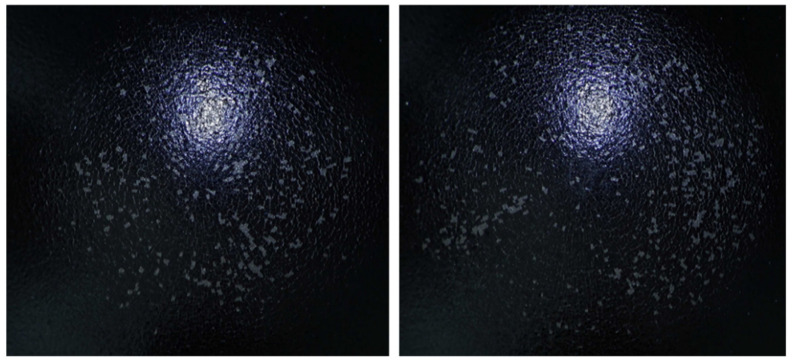
Partially Damaged Sample (Factor 0).

**Figure 18:**
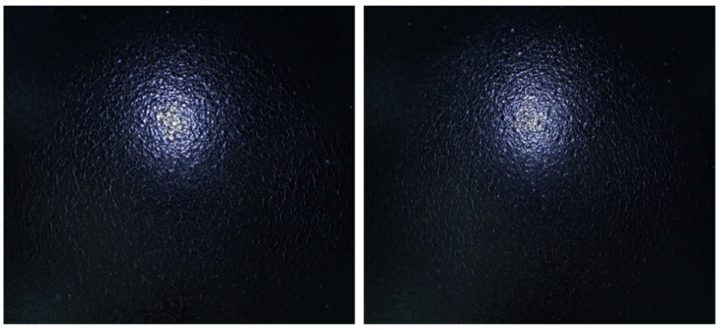
Undamaged Sample (Factor 1).

These parameters define the reproducible dynamic load conditions under which the coatings were systematically examined, ensuring consistent and comparable testing across all samples. Damage evaluation was performed using an INSPECTIS digital optical system, which allows for high-resolution visualization and precise documentation of the impacted area, facilitating detailed analysis of microstructural changes and surface deformation patterns (Figure 16, Figure 17 and Figure 18). The extent of surface failure was subsequently classified according to three discrete categories, enabling a standardized and quantitative assessment of coating integrity under dynamic mechanical loading:

- Factor −1: Extensive damage—pronounced cracking and delamination

- Factor 0: Partial damage—minor cracks present but no significant delamination

- Factor 1: Minimal or no visible damage, with the coating structure intact.

Representative images presented in Figure 16, Figure 17 and Figure 18, corresponding to the distinct damage levels (Factor −1, Factor 0, Factor 1) within the classification system, were applied to evaluate the influence of curing conditions on the mechanical integrity of the coatings. Table 12 and Table 13 summarize the distribution of damage factors as a function of curing time and curing temperature, respectively. Shorter curing durations were associated with a higher prevalence of Factor −1, indicating insufficient consolidation of the polymer network and reduced impact resistance. Conversely, extended curing times resulted in coatings predominantly assigned to Factor 1, reflecting enhanced structural integrity. A similar trend was observed for curing temperature: insufficient thermal treatment promoted cracking and delamination, whereas optimal thermal curing led to undamaged surfaces (Factor 1).

**Figure 19:**
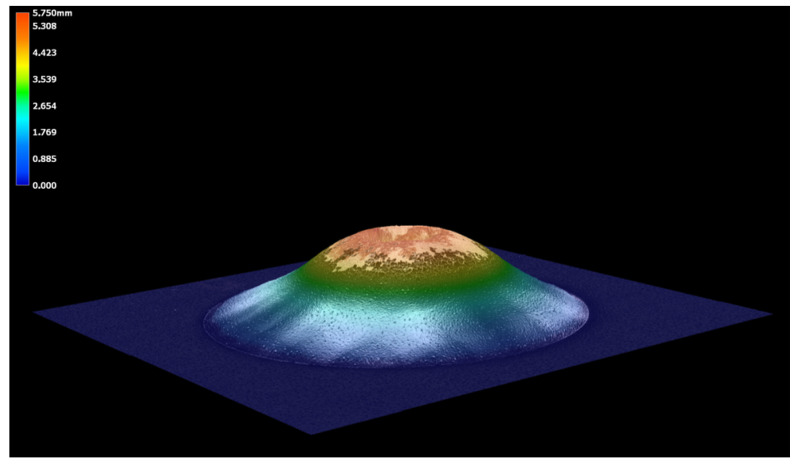
Three-dimensional surface profile of impact-induced damage.

The three-dimensional profilometric reconstruction of the impact site, obtained using the Keyence Polarize digital optical system, provides a detailed visualization of the coating damage induced by the standardized falling-weight test, as shown in Figure 19.

The analysis revealed that the deformation depth ranged from 0.564 mm to 5.750 mm, indicating severe local disruption of the coating–substrate system under impact loading. The values were obtained using the Keyence Polarize system, which enabled precise monitoring of the maximum load response by recording the depth of indentation caused by the spherical indenter. The impact test was performed on each specimen in four repetitions, ensuring reproducibility and verification of measurement reliability. Accordingly, for the factor X_1_—influence of curing time on impact resistance—a total of 20 individual impact tests were carried out (four per specimen), and for the factor X_2_—influence of curing temperature on impact resistance—another 20 impact tests were conducted under the same conditions. The recorded depths confirmed that the extent of coating damage varied significantly across the tested samples. As shown in Figure 19, Sample n. 5 from the X_2_ experiment exhibited the highest deformation depth of 5.750 mm, representing the maximum damage observed in the entire DoE. The crater-shaped morphology with a steep central indentation and concentric plastic flow zones corresponds to a Factor −1 classification, characterized by extensive cracking and delamination. This observation corroborates the classification framework applied in Table 12 and Table 13, where insufficient curing conditions resulted in a predominance of Factor −1 failures. The magnitude of the measured indentation depth thus reflects the inability of inadequately consolidated coatings to dissipate the imparted impact energy (39.2 J) without structural breakdown.

Conversely, specimens subjected to optimized curing conditions (Factor 1) demonstrated significantly reduced or absent surface disruption, highlighting the crucial role of crosslinking kinetics in providing resistance to dynamic mechanical loading. Taken together, the profilometric analysis not only confirms the qualitative classification of damage severity but also provides direct evidence of the mechanical fragility associated with suboptimal curing. Overall, the results demonstrate that the applied impact test is a robust method for evaluating the resistance of cathodic electrodeposited coatings to rapid deformation. The findings further confirm that curing time and temperature are critical parameters governing the coating’s ability to withstand dynamic mechanical stress.

### 3.4. Integrated Evaluation of Mechanical Performance

To reduce the subjectivity inherent in conventional classification methods and enable a more objective interpretation of coating performance, the results of adhesion and impact testing were quantitatively transformed. Adhesion ratings obtained in accordance with ISO 2409:2020 were converted into relative percentage values of adhesion integrity (Grade 0 = 100%, Grade 1 = 80%, Grade 2 = 60%, etc.). Likewise, damage factors determined from impact testing (ISO 6272-1:2011) were expressed as integrity percentages (Factor 1 = 100%, Factor 0 = 50%, Factor −1 = 0%).

In parallel with qualitative classification, the physical extent of surface damage was assessed through crater depth measurement. The deviation did not exceed ±0.05 mm for any of the specimens, indicating no statistically significant geometric variation. Consequently, the analysis focused primarily on the coating’s failure response rather than on crater morphology.

#### 3.4.1. Effect of Curing Time on Mechanical Performance

Table 14 summarizes the influence of curing time on adhesion and impact resistance, presenting ISO grades, transformed integrity values, damage factors, crater depth, and mean coating thickness. Adhesion improved from 80% at 13 min to a peak of 100% at 17–23 min, followed by a decline to 80% at 27 min. A parallel trend was found for impact integrity, reaching its maximum at 20 min.

The crater depth remained stable (±0.05 mm), confirming that differences in coating response were driven by material behavior rather than by test geometry. Average film thickness varied between 14.6 µm and 18.3 µm without a clear correlation to curing time. This indicates that variations in performance were governed primarily by the degree of polymerization and internal stress development.

**Figure 20:**
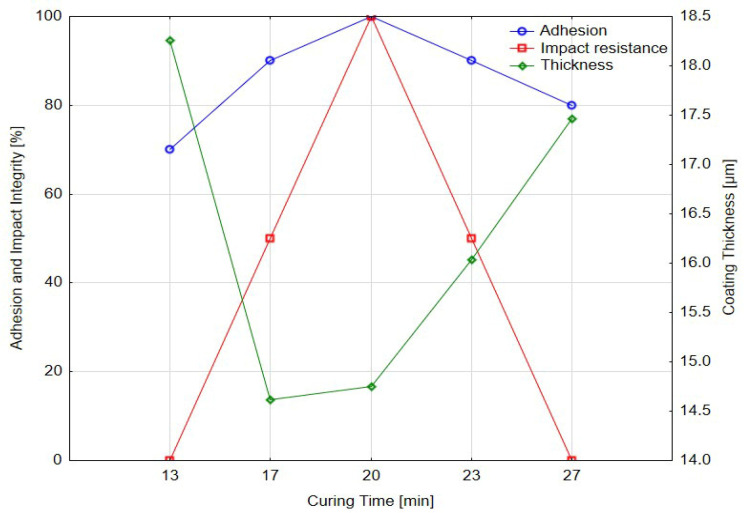
Effect of curing time [min] on adhesion integrity, impact resistance and coating thickness.

Figure 20 shows the evolution of adhesion, impact resistance and coating thickness with curing time. The optimum performance was achieved at 20 min, corresponding to balanced interfacial bonding and cohesive strength. Insufficient curing (13 min) led to reduced adhesion and complete impact failure, while over-curing (27 min) caused a loss of flexibility and reduced impact integrity. Thickness remained essentially stable, supporting the conclusion that mechanical behavior was dictated by chemical and structural processes rather than film geometry.

#### 3.4.2. Effect of Curing Temperature on Mechanical Performance

Table 15 presents the results of adhesion and impact testing as a function of curing temperature. Adhesion integrity rose from 80% at 150 °C to 90% at 176 °C, then decreased again to 80% at 250 °C. The highest impact integrity (100%) occurred at 200 °C, indicating optimal toughness and interfacial stability at this curing temperature. At 176 °C and 250 °C, however, the coating exhibited complete failure during impact testing, suggesting inadequate or excessive crosslinking and a loss of ductility.

Coating thickness ranged from 15.7 µm to 18.5 µm, and crater depth remained effectively constant, reinforcing that temperature-induced changes in mechanical behavior stemmed from polymer network evolution rather than geometric effects.

**Figure 21:**
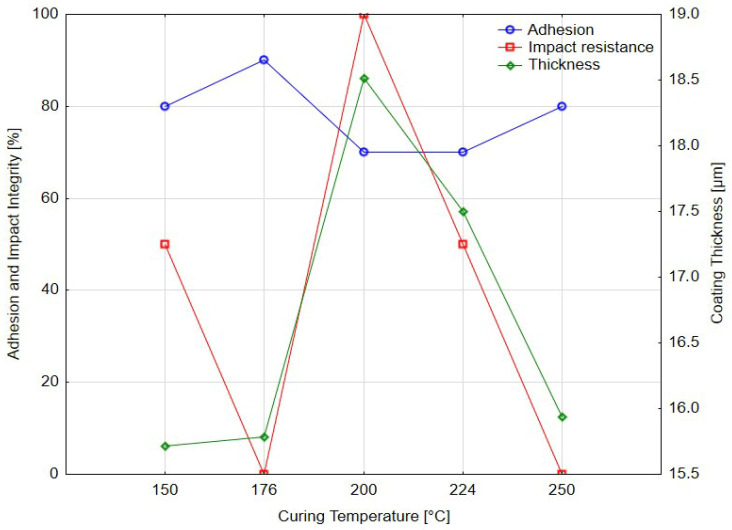
Effect of curing temperature [°C] on adhesion integrity, impact resistance and coating thickness.

Figure 21 depicts the relationship between curing temperature and coating performance. Optimal behavior was recorded at 200 °C, where both adhesion and impact resistance peaked, supported by slightly increased thickness. Lower temperatures (150 °C) resulted in incomplete curing and reduced impact performance—the coating does not achieve full crosslinking, resulting in incomplete adhesion and reduced impact resistance—while higher temperatures (≥250 °C) led to embrittlement and diminished resistance to excessive hardening, increased internal stress and a corresponding reduction in impact integrity despite maintaining acceptable adhesion values.

### 3.5. Elemental Composition Analysis (EDX)

To verify the elemental composition and structural integrity of the cathodic electrodeposited (CED) coatings, a detailed EDX analysis was performed on the cross-section of the coated VDA-239 CR4 steel substrate. The analysis was conducted on Sample n.3 (X_2_), which represented the optimal curing conditions identified during the experimental evaluation of curing parameters. The purpose of this analysis was to confirm the compositional consistency and microstructural integrity of the coating formed under these optimized curing conditions.

**Figure 22:**
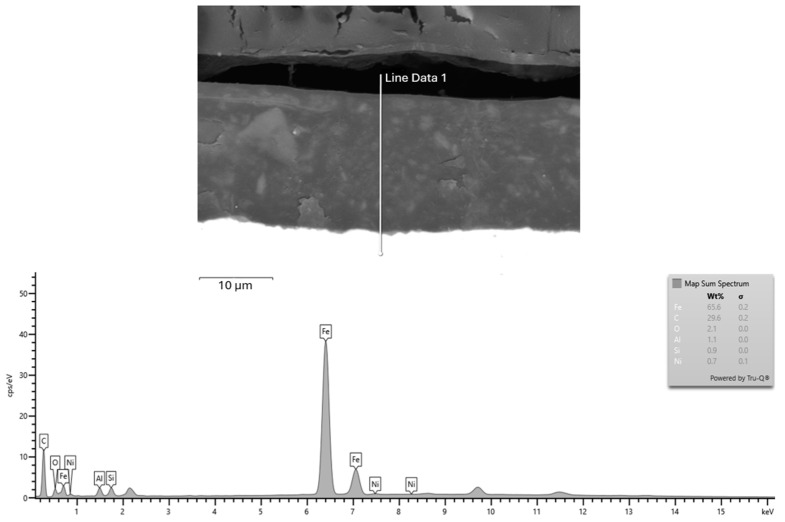
SEM and Elemental Composition Analysis (EDX).

Figure 22 shows the SEM micrograph and corresponding EDX spectrum obtained from the interface region (Line Data 1). The results confirm that the coating primarily consists of iron (Fe ≈ 65.6 wt%) originating from the substrate, and carbon (C ≈ 29.6 wt%) representing the organic polymeric matrix formed during electrodeposition. Minor constituents, including oxygen (O ≈ 2.1 wt%), aluminum (Al ≈ 1.1 wt%), silicon (Si ≈ 0.9 wt%), and nickel (Ni ≈ 0.7 wt%), were also detected, indicating the presence of residual elements from surface pretreatment and alloying. The SEM cross-section demonstrates a continuous and compact interface between the coating and the steel substrate, without signs of delamination or porosity. This confirms that the applied curing regime provided sufficient activation energy for polymer crosslinking and interfacial adhesion.

**Figure 23:**
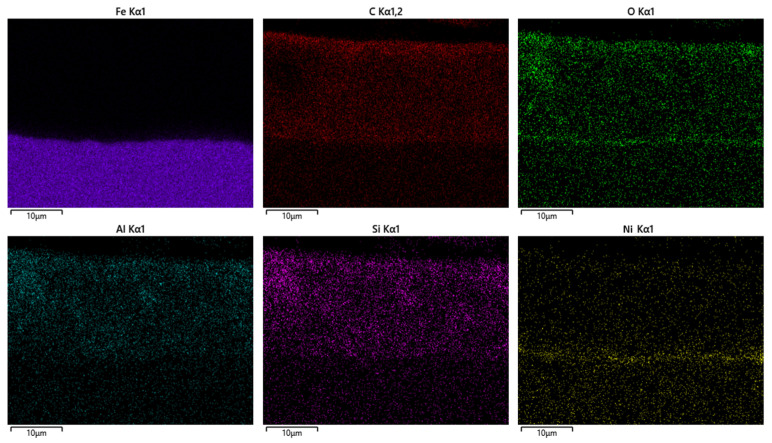
EDX elemental mapping of the CED coating cross-section.

To visualize the spatial distribution of individual elements within the CED coating, EDX elemental mapping was performed across the cross-section (Figure 23). The mapping results confirm the compositional stratification of Fe, C, O, Al, Si and Ni and interfacial continuity of the coating system.

The carbon (C Kα_1_) map shows a uniform distribution throughout the coating thickness, verifying the continuity of the polymeric organic matrix formed during the cathodic deposition and curing processes. Oxygen (O Kα_1_) is distributed relatively evenly across the layer, indicating stable oxidation states and uniform crosslinking reactions within the polymer network. The presence of aluminum (Al Kα_1_) and nickel (Ni Kα_1_) is localized near the interface between the coating and the steel substrate, corresponding to residues from phosphate-based pretreatment layers that enhance adhesion and corrosion resistance. The iron (Fe Kα_1_) signal is concentrated exclusively within the substrate region, confirming the compactness of the coating and the absence of metal diffusion into the polymer layer. Similarly, silicon (Si Kα_1_) appears as a minor constituent distributed near the surface, likely associated with filler particles or surface contaminants incorporated during deposition.

**Figure 24:**
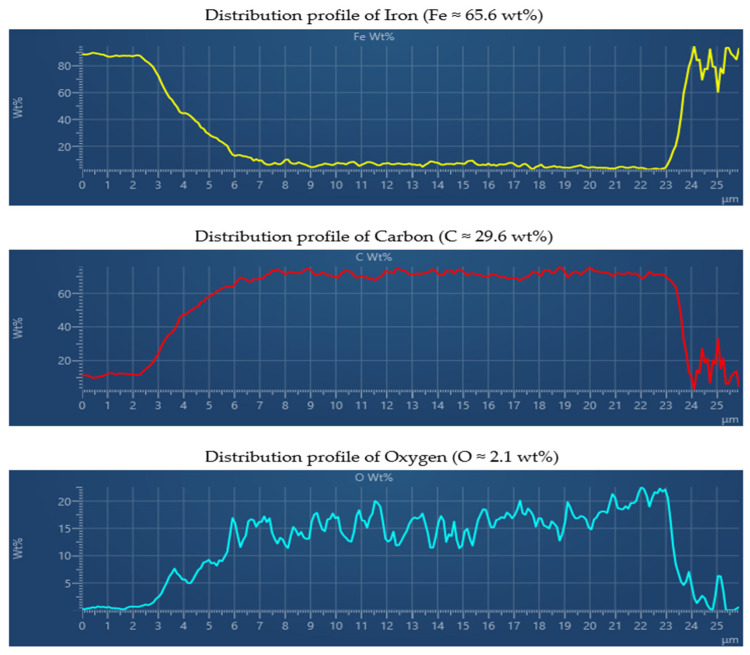
EDX line scan analysis of the CED coating—Fe, C and O.

To gain further insight into the depth-dependent chemical composition, EDX line-scan analyses were carried out along the cross-section of the CED coating. Figure 24 presents the results of the line scan focusing on the primary elements Fe, C, and O. The iron (Fe Kα_1_) concentration profile exhibits a sharp and well-defined transition between the metallic substrate and the organic coating, confirming the absence of metallic diffusion into the polymeric matrix. The Fe signal remains constant within the substrate and decreases steeply at the interface, demonstrating the compactness of the coating and the effective formation of a barrier preventing substrate element penetration. Conversely, the carbon (C Kα_1_) profile shows a homogeneous distribution throughout the coating thickness, reflecting uniform formation of the organic matrix during electrophoretic deposition and subsequent curing. The oxygen (O Kα_1_) signal is evenly distributed within the coating, with a slight increase near the surface, corresponding to oxidation and crosslinking reactions occurring during curing. This behavior confirms that the coating achieved the desired microstructural homogeneity, which is critical for ensuring its barrier and adhesion performance.

**Figure 25:**
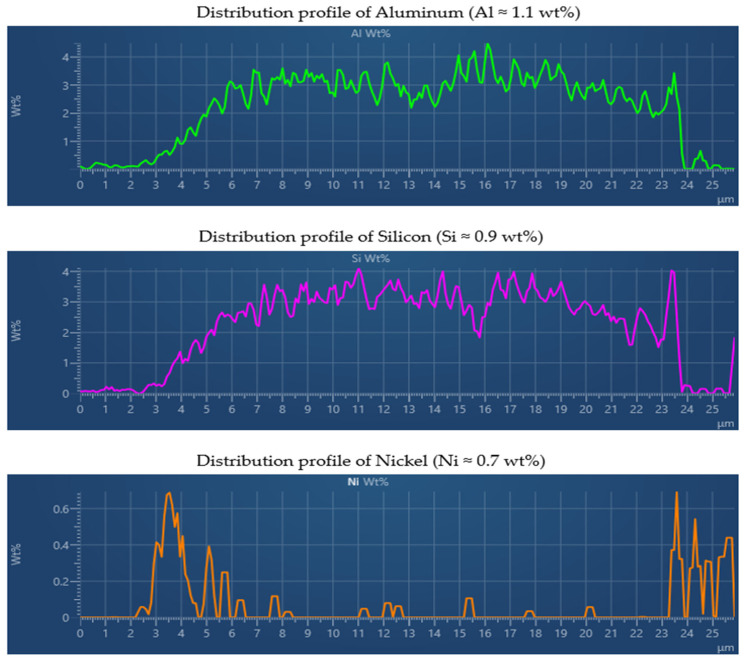
EDX line scan analysis of the CED coating—Al, Si and Ni.

In addition to the primary elements, the distribution of secondary alloying and pretreatment elements was examined to better understand the interfacial chemistry (Figure 25). Aluminum (Al Kα_1_) and nickel (Ni Kα_1_) exhibit clear concentration peaks within a narrow zone at the interface between the substrate and the polymer layer. This interfacial accumulation is consistent with the use of phosphate or zirconium-based pretreatments that enhance coating adhesion and corrosion resistance. Silicon (Si Kα_1_) displays a low but detectable concentration, predominantly near the surface layer, most likely due to the presence of filler particles or residual contaminants from the processing environment.

These results are in line with reported studies that highlight the importance of high-resolution elemental mapping for understanding material performance. Researcher Tromp [31] demonstrated that integrating EDX with low-energy electron microscopy enables precise spatial resolution of elemental distributions in nanostructured systems, which is conceptually similar to our observation of well-defined interfacial enrichment in the CED coatings. Moreover, Researcher Qi et al. [32] applied wide-field EDX absorption spectroscopy to heterogeneous biological samples, emphasizing how controlled elemental distribution correlates with functional stability—a principle that mirrors our findings of uniform C and O profiles, ensuring effective curing and film formation. Finally, Researcher Nam et al. [33] showed that compositional uniformity in Cu_2_ZnSnSe_4_ thin films strongly influences structural integrity and functional performance, supporting our conclusion that chemical homogeneity in the CED coatings directly contributes to adhesion, reproducibility and corrosion protection. Collectively, these studies validate that precise elemental control, whether at the nano or microscale, underpins the functional reliability of multilayered coatings, reinforcing the significance of our findings for industrial electrodeposition processes.

The combined results from SEM–EDX spectrum, elemental mapping, and line-scan analyses (Figure 22, Figure 23, Figure 24 and Figure 25), performed on the reference sample n.3 (X_2_ variable), which exhibited the highest average coating thickness (18.517 µm) and optimal mechanical performance, clearly confirm that the CED coating produced under optimized curing conditions exhibits a chemically stable and structurally compact configuration. The homogeneous distribution of carbon and oxygen within the organic matrix, together with localized interfacial enrichment of aluminum and nickel, validates the efficiency of the polymerization and crosslinking processes. The Fe, C, and O line profiles demonstrated a distinct boundary between the metallic substrate and the polymeric film, confirming the absence of elemental diffusion and the formation of a well-defined barrier layer. Additional elements such as Al, Si, and Ni, detected in minor concentrations, were confined to the interfacial zone, reflecting the effect of phosphate-based pretreatment and supporting enhanced interfacial adhesion. These microanalytical findings are fully consistent with the mechanical testing results, where the reference sample reached ISO adhesion Grade 1–2 and full impact integrity (100%), and with the statistical capability indices obtained in PalstatCAQ (Cp = 2.29; Pp = 1.44). The correlation between EDX compositional uniformity, mechanical performance, and process capability parameters provides a solid experimental foundation linking chemical stability, interfacial quality, and manufacturing reproducibility in cathodic electrodeposition systems.

## 4. Conclusions

The research introduced a novel integrated approach to evaluating anticorrosive CED coatings by combining non-destructive and destructive testing with statistical process capability analysis. Unlike conventional studies, this work demonstrates the simultaneous application of standardized thickness, adhesion, and impact resistance tests with Cp, Cpk, Pp, and Ppk indices, providing a comprehensive assessment of both coating performance and process robustness.

From a technical perspective, the results highlight several key findings:

- Coating thickness exhibited non-linear dependencies on curing parameters, with shorter curing times producing mean values up to 18.3 µm, while both under- and over-curing reduced thickness stability.
- Capability indices indicated sufficient potential capability (Cp = 2.29; Pp = 1.44) but significantly lower real performance (Cpk = 0.34; Ppk = 0.22), confirming systematic mean shifts and deviations from normality as verified by Shapiro–Wilk and Anderson–Darling tests.
- Adhesion tests according to ISO 2409:2020 demonstrated a strong correlation between curing conditions and interfacial bonding, with optimally polymerized samples achieving Grade 0 classification.
- Impact resistance analysis per ISO 6272-1:2011 revealed that insufficient curing resulted in extensive cracking and delamination (Factor −1), whereas optimized curing provided intact coatings (Factor 1), supported by profilometric evidence of reduced indentation depth.

Additionally, the EDX analysis of Sample n.3 (X_2_) confirmed the chemical integrity and multilayered composition of the CED coating. The homogeneous distribution of carbon and oxygen within the polymeric matrix, combined with localized enrichment of aluminum and nickel at the metal–polymer interface, validated the effectiveness of the applied curing regime. These findings substantiate that optimized thermal curing not only enhances coating adhesion and compactness but also stabilizes the interfacial chemistry, ensuring reliable corrosion protection and long-term performance.

The novelty of the study lies in the integration of coating performance testing with advanced capability analysis, bridging material characterization with statistical quality control in compliance with ISO 22514-7:2021 and IATF 16949:2016. From an industrial standpoint, the results emphasize that precise control of curing time and temperature is critical not only for achieving the required coating integrity but also for ensuring reproducible process capability in automotive and mechanical engineering applications.

Future research should expand upon these findings by addressing additional process variables that were not within the scope of this study, such as bath composition, pH stability, and electrode geometry, which are expected to exert significant influence on coating microstructure and long-term performance. Moreover, the integration of advanced in situ monitoring techniques, including electrochemical impedance spectroscopy (EIS) and real-time thermal analysis, could provide deeper mechanistic insights into polymerization kinetics and stress development during curing. Another promising direction involves the incorporation of nanostructured fillers or hybrid coatings, which may enhance barrier properties and improve resistance to localized corrosion. From a methodological perspective, further refinement of process capability analysis through machine-learning-based predictive modeling could strengthen the link between statistical indices and coating functionality, enabling real-time process control in industrial environments. Finally, long-term durability studies under cyclic environmental and mechanical loading should be conducted to validate the predictive framework established in this work and ensure its applicability in automotive sectors.

## Figures and Tables

**Table 1:** Mechanical properties of VDA 239-100 CR4 [20].

Property	Yield Strength (Rp0.2)	Tensile Strength (Rm)	Brinell Hardness (HBW)	Elongation	r-Value (r 90/20)	r-Value (r m/20)	n 10–20/Ag
Value	140–180 MPa	140–180 MPa	267	≥39%	≥1.9	≥1.6	≥0.20

**Table 2:** Chemical properties of VDA 239-100 CR4 [20].

Element	C Max.	Mn Max.	Si Max.	P Max.	S Max.	Al Min.	Ti Max.	Cu Max.
CR4 [%]	0.06	0.40	0.50	0.025	0.020	0.010	0.30	0.20

**Table 3:** Technological sequence of cathodic electrodeposition coating.

Process No.	Step	Technological Operation
1	1	Chemical degreasing—stage 1
2	2	Chemical degreasing—stage 2
3	3.1	Rinsing in demineralized water—stage 1
4	3.2	Rinsing in demineralized water—stage 2
5	4	Surface activation prior to phosphating
6	5	Phosphating
7	6.1	Rinsing in demineralized water—stage 1
8	6.2	Rinsing in demineralized water—stage 2
9	7	Cataphoretic electrodeposition (KTL)
10	8.1–8.2	Rinsing in ultrafiltrate
11	9.1–9.3	Curing

**Table 4:** Controlled input factors of the planned experimental methodology.

Factor Code	Variable	Symbol	−2.05464	−1	0	1	2.05464
X_1_	Curing— Deposition time	[min]	13	17	20	23	27
X_2_	Curing— Temperature	[°C]	150	176	200	224	250

**Table 5:** Constant factors of the planned experimental methodology.

Factor Code	Variable	Symbol	Value
X_3_	Degreasing solution	[g·L^−1^]	35
X_4_	Chemical degreasing—deposition time	[min]	8
X_5_	Degreasing temperature	[°C]	60
X_6_	Phosphating—deposition time	[min]	5
X_7_	CED—deposition time	[min]	5
X_8_	CED—voltage	[V]	250

**Table 6:** Effect of Curing Time [min] on Coating Thickness [µm].

Descriptive Statistics of Curing Time—Layer Thickness [μm]					
Variable X_1_	Valid N	Mean	Minimum	Maximum	Std. Dev
Sample n.1 (X_1_)	30	18.25667	15.30000	21.30000	1.454999
Sample n.2 (X_1_)	30	14.61667	12.90000	16.70000	0.982637
Sample n.3 (X_1_)	30	14.75000	12.20000	17.20000	1.177593
Sample n.4 (X_1_)	30	16.03667	14.00000	18.60000	1.306874
Sample n.5 (X_1_)	30	17.46667	14.70000	20.10000	1.269628

**Table 7:** Process Stability and Performance According to ISO 22514 under Effects of Curing Time.

L	Index	X (99.865–0.135%) Fit	X (99.865–0.135%) Real	√(s^2^)	s′/c_4_	R′/d_2_	σ
L1—X′	Pp/Cp	1.435	2.032	2.287	2.287	2.254	1.435
	Ppk/Cpk	0.216	0.357	0.344	0.345	0.339	0.216
	Ppkl/Cpkl	0.216	0.357	0.344	0.345	0.339	0.216
	Ppku/Cpku	2.654	3.287	4.230	4.230	4.169	2.654
L2—X∿	Pp/Cp	1.435	2.032	2.287	2.287	2.254	1.435
	Ppk/Cpk	0.163	0.282	0.244	0.244	0.24	0.163
	Ppkl/Cpkl	0.163	0.282	0.244	0.244	0.24	0.163
	Ppku/Cpku	2.556	3.121	4.230	4.230	4.169	2.717
L3—X″	Pp/Cp	1.435	2.032	2.287	2.287	2.254	1.435
	Ppk/Cpk	0.216	0.357	0.344	0.345	0.339	0.216
	Ppkl/Cpkl	0.216	0.357	0.344	0.345	0.339	0.216
	Ppku/Cpku	2.654	3.287	4.230	4.230	4.169	2.654
L4—X∿′	Pp/Cp	1.435	2.032	2.287	2.287	2.254	1.435
	Ppk/Cpk	0.202	0.338	0.317	0.317	0.313	0.199
	Ppkl/Cpkl	0.202	0.338	0.317	0.317	0.313	0.199
	Ppku/Cpku	2.626	3.239	4.257	4.258	4.195	2.671

**Table 8:** Effect of Curing Temperature [°C] on Coating Thickness [µm].

Descriptive Statistics of Curing Temperature—Layer Thickness [μm]					
Variable X_1_	Valid N	Mean	Minimum	Maximum	Std. Dev
Sample n.1 (X_2_)	30	15.71333	13.50000	18.90000	1.185758
Sample n.2 (X_2_)	30	15.78667	13.50000	20.80000	1.447169
Sample n.3 (X_2_)	30	18.51667	15.90000	24.10000	1.849899
Sample n.4 (X_2_)	30	17.49667	14.90000	21.30000	1.513499
Sample n.5 (X_2_)	30	15.94000	14.00000	18.10000	0.959741

**Table 9:** Process Stability and Performance According to ISO 22514 under Effects of Curing Temperature.

L	Index	X (99.865–0.135%) Fit	X (99.865–0.135%) Real	√(s^2^)	s′/c_4_	R′/d_2_	σ
L1—X′	Pp/Cp	1.407	1.466	1.804	1.851	1.724	1.407
	Ppk/Cpk	0.314	0.538	0.402	0.413	0.384	0.314
	Ppkl/Cpkl	0.314	0.538	0.402	0.413	0.384	0.314
	Ppku/Cpku	2.500	1.870	3.206	3.289	3.064	2.500
L2—X∿	Pp/Cp	1.407	1.466	1.804	1.851	1.724	1.407
	Ppk/Cpk	0.247	0.456	0.289	0.296	0.276	0.225
	Ppkl/Cpkl	0.247	0.456	0.289	0.296	0.276	0.225
	Ppku/Cpku	2.378	1.870	3.206	3.289	3.172	2.378
L3—X″	Pp/Cp	1.407	1.466	1.804	1.851	1.724	1.407
	Ppk/Cpk	0.314	0.538	0.402	0.413	0.384	0.314
	Ppkl/Cpkl	0.314	0.538	0.402	0.413	0.384	0.314
	Ppku/Cpku	2.500	1.870	3.206	3.289	3.064	2.500
L4—X∿′	Pp/Cp	1.407	1.466	1.804	1.851	1.724	1.407
	Ppk/Cpk	0.296	0.518	0.37	0.38	0.354	0.289
	Ppkl/Cpkl	0.296	0.518	0.37	0.38	0.354	0.289
	Ppku/Cpku	2.464	1.854	3.237	3.321	3.094	2.525

**Table 10:** Influence of Curing Time on cross-cut test results.

Effect of Curing Time [min] on Coating Adhesion				
Variable X_1_	Grid A	Grid B	Grid C	Grid D
Sample n.1 (X_1_)	1	1	2	1
Sample n.2 (X_1_)	1	0	1	0
Sample n.3 (X_1_)	1	0	1	0
Sample n.4 (X_1_)	0	0	1	1
Sample n.5 (X_1_)	1	1	1	1

**Table 11:** Influence of Curing Temperature on cross-cut test results.

Effect of Curing Temperature [°C] on Coating Adhesion				
Variable X_2_	Grid A	Grid B	Grid C	Grid D
Sample n.1 (X_2_)	1	0	1	1
Sample n.2 (X_2_)	0	1	0	1
Sample n.3 (X_2_)	1	1	2	1
Sample n.4 (X_2_)	1	0	1	2
Sample n.5 (X_2_)	1	0	1	1

**Table 12:** Influence of curing time on impact resistance.

Effect of Curing Time [min] on Coating Impact Resistance			
Variable X_1_	−1	0	1
Sample n.1 (X_1_)	✓	-	-
Sample n.2 (X_1_)	-	✓	-
Sample n.3 (X_1_)	✓	-	-
Sample n.4 (X_1_)	-	✓	-
Sample n.5 (X_1_)	✓	-	-

**Table 13:** Influence of curing temperature on impact resistance.

Effect of Curing Temperature [°C] on Coating Impact Resistance			
Variable X_2_	−1	0	1
Sample n.1 (X_2_)	-	✓	-
Sample n.2 (X_2_)	✓	-	-
Sample n.3 (X_2_)	-	-	✓
Sample n.4 (X_2_)	-	✓	-
Sample n.5 (X_2_)	✓	-	-

**Table 14:** Effect of Curing Time [min] on Mechanical Performance.

Variable X_1_	Curing Time [min]	ISO Grade	Adhesion Integrity [%]	Damage Factor	Impact Integrity [%]	Mean Thickness [µm]
Sample n.1 (X_1_)	13	1~2	80%~60%	−1	0%	18.257
Sample n.2 (X_1_)	17	0~1	100%~80%	0	50%	14.617
Sample n.3 (X_1_)	20	0~1	100%~80%	−1	0%	14.750
Sample n.4 (X_1_)	23	0~1	100%~80%	0	50%	16.037
Sample n.5 (X_1_)	27	1	80%	−1	0%	17.467

**Table 15:** Effect of Curing Temperature [°C] on Mechanical Performance.

Variable X_2_	Temperature [°C]	ISO Grade	Adhesion Integrity [%]	Damage Factor	Impact Integrity [%]	Mean Thickness [µm]
Sample n.1 (X_2_)	150	1	80%	0	50%	15.713
Sample n.2 (X_2_)	176	0~1	100%~80%	−1	0%	15.787
Sample n.3 (X_2_)	200	1~2	80%~60%	1	100%	18.517
Sample n.4 (X_2_)	224	1~2	80%~60%	0	50%	17.497
Sample n.5 (X_2_)	250	1	80%	−1	0%	15.940

## Data Availability

The original contributions presented in the study are included in the article, further inquiries can be directed to the corresponding authors.
